# Foot‐and‐Mouth Disease Impact on Smallholders ‐ What Do We Know, What Don't We Know and How Can We Find Out More?

**DOI:** 10.1111/tbed.12507

**Published:** 2016-05-11

**Authors:** T. J. D. Knight‐Jones, M. McLaws, J. Rushton

**Affiliations:** ^1^ International Livestock Research Institute [ILRI] Lusaka Zambia; ^2^ European Commission for the Control of FMD FAO Rome Italy; ^3^ The Royal Veterinary College (VEEPH) North Mymms Hertfordshire UK

**Keywords:** Foot‐and‐mouth disease, smallholder, economics, impact, serology, seroprevalence

## Abstract

Foot‐and‐mouth disease (FMD) endemic regions contain three‐quarters of the world's FMD susceptible livestock and most of the world's poor livestock keepers. Yet FMD impact on smallholders in these regions is poorly understood. Diseases of low mortality can exert a large impact if incidence is high. Modelling and field studies commonly find high FMD incidence in endemic countries. Sero‐surveys typically find a third of young cattle are sero‐positive, however, the proportion of sero‐positive animals that developed disease, and resulting impact, are unknown. The few smallholder FMD impact studies that have been performed assessed different aspects of impact, using different approaches. They find that FMD impact can be high (>10% of annual household income). However, impact is highly variable, being a function of FMD incidence and dependency on activities affected by FMD. FMD restricts investment in productive but less FMD‐resilient farming methods, however, other barriers to efficient production may exist, reducing the benefits of FMD control. Applying control measures is costly and can have wide‐reaching negative impacts; veterinary‐cordon‐fences may damage wildlife populations, and livestock movement restrictions and trade bans damage farmer profits and the wider economy. When control measures are ineffective, farmers, society and wildlife may experience the burden of control without reducing disease burden. Foot‐and‐mouth disease control has benefitted smallholders in South America and elsewhere. Success takes decades of regional cooperation with effective veterinary services and widespread farmer participation. However, both the likelihood of success and the full cost of control measures must be considered. Controlling FMD in smallholder systems is challenging, particularly when movement restrictions are hard to enforce. In parts of Africa this is compounded by endemically infected wildlife and limited vaccine performance. This paper reviews FMD impact on smallholders in endemic countries. Significant evidence gaps exist and guidance on the design of FMD impact studies is provided.

## Introduction

**Figure 1:**
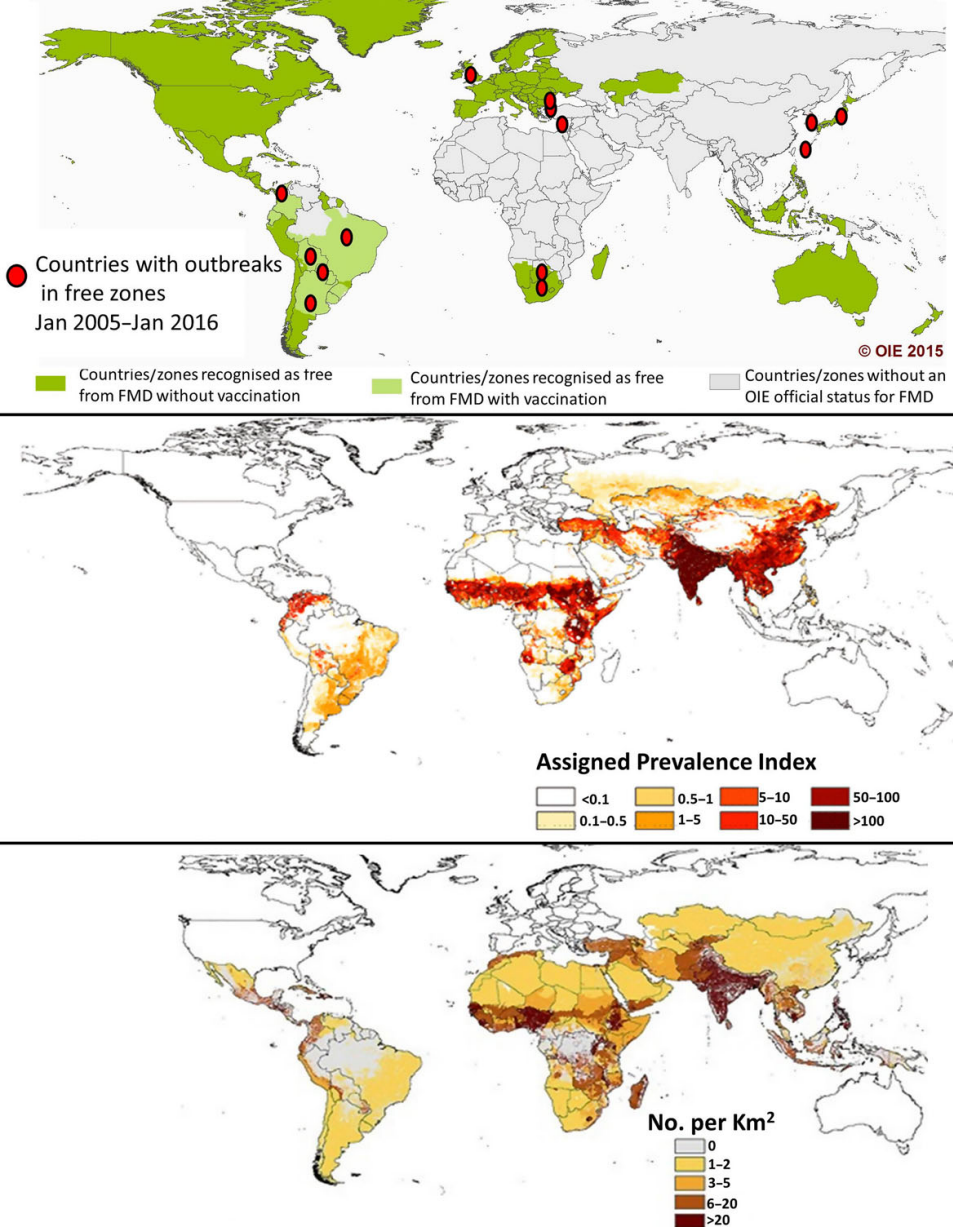
Upper panel – May 2015 OIE global FMD status showing outbreaks in FMD‐free countries/zones that occurred between Jan 2005 and Jan 2016 ‐ map adapted from OIE FMD status map extracted 4th April 2016 from http://www.oie.int/en/animal-health-in-the-world/official-disease-status/fmd/en-fmd-carte/. Middle panel – global burden of FMD in cattle in 2008 (burden in sheep and goats has a similar distribution). Prevalence index based on estimates of incidence, population distribution and other risk factors, adapted from (Sumption et al., 2008). Note progress in South America since 2008 [compare with upper panel]. Lower panel – density of poor rural livestock keepers updated from Thornton et al. (2002). Central America, zones in Kazakhstan and Southern Africa, parts of South East Asia and some areas of South America are among the few exceptions where FMD is not present in poor livestock keeper populations.

The global burden of Foot‐and‐mouth disease (FMD) mirrors the distribution of poor livestock keepers (Fig. 1) (Rushton and Knight‐Jones, 2012). Little has been done to quantify this burden. Although the dramatic impact of FMD outbreaks in countries where the disease has been eradicated is well‐understood (global costs of approximately US$1.5 billion per year), less is known about impact in countries where the virus is endemic, even though FMD impact is likely to be far greater in endemic regions (estimated global costs of >$6.5 billion a year resulting from disease and vaccination alone) (Knight‐Jones and Rushton, 2013; Robinson and Knight‐Jones, 2014).

The impact of FMD on smallholders has been particularly neglected. A review of FMD in Southern Africa (Thomson, 1995) stated that ‘FMD has its major effect on intensively farmed, high‐producing livestock and…when it occurs in [small‐scale commercial agriculture or extensive sectors] its direct effect…is usually limited…, although this has not been specifically investigated’. As it has not been adequately investigated, the impact of FMD on smallholder farmers in regions where the virus is endemic remains uncertain and is often contested (Scoones and Wolmer, 2007; Perry and Grace, 2009; Perry and Rich, 2007).

Other diseases have much higher mortality rates than FMD, however, a common disease with low mortality can still exert a heavy burden on a population. A failure to appreciate this population‐level burden may be compounded by under‐reporting of cases of disease; as is the case for influenza and many food‐borne diseases in humans (Mead et al., 1999; Reed et al., 2015). The impact of FMD is complex, with direct and indirect impacts, as well as visible and invisible losses; all can be substantial, difficult to estimate and highly variable (Knight‐Jones and Rushton, 2013).

An understanding of disease impact is needed to guide livestock disease control policy. In fact some FMD‐endemic countries invest large amounts in FMD control whereas others invest little to nothing. Often policy decisions are made without adequate consideration of the economic impact of the disease and its control. This may be due to a lack of awareness of what is known about FMD impact on smallholder systems in endemic countries, the lack of studies of the subject and the lack of guidance on how to assess it.

Here, we present findings from a review of FMD impact, specifically focussing on smallholders in regions where the virus is endemic. This builds on previous work by the authors that looked at global FMD impact and its complexities (Knight‐Jones and Rushton, 2013; Rushton and Knight‐Jones, 2012). Although the focus is on Africa and Asia, we explore universal knowledge gaps, and consider requirements for impact studies, and the strengths and weaknesses of different approaches.

## Approach

### Literature review

A literature search was conducted reviewing published journal articles, reports and grey literature. The search used the following methods:

1. Online search: Pubmed, google scholar and google web were searched for papers containing ‘FMD’ or ‘foot and mouth disease’ and ‘economic*’ or ‘impact’ or ‘cost‐benefit’; as well as ‘aphteuse’ and ‘impact’ or ‘économique’.
2. Experts from 14 groups working in the field of FMD economics were asked to provide suitable publications and also to suggest other experts to contact (see Table S1 for the list of experts engaged).

Papers in English, Spanish and French were included in the review. Articles were retained if they reported research on FMD economic impact.

### What is a smallholder?

Many pastoralists maintain large herds (Jabra, 2010). Despite this, many are economically vulnerable, with low indicators of economic well‐being (health, education, income). Hence, in this article ‘smallholder’ refers to an economically vulnerable household whose income significantly depends upon FMD‐susceptible livestock (principally cattle, water buffalo, goats, sheep and pigs) and includes pastoralists and agro‐pastoralists.

## FMD smallholder impact: what do we know and what don't we know?

Identified papers that are relevant to this study are listed in the ‘review bibliography’ included in the Electronic Supplementary Material (ESM Appendix S2) and are separated into the categories (i) General; (ii) Africa and (iii) Asia.

### Impact estimation

#### Overview of the literature

Despite the ‘urgent need’ for more studies of FMD impact on the poor (Perry and Grace, 2009), relatively little has been done. Table 1 provides more details of FMD impact studies from different countries. Although they highlight many different aspects of FMD impact, the studies cannot easily be compared due to differences in objectives and approach, however, impact appears to vary by region, agro‐ecological setting and production system. No comprehensive economic welfare analysis has been performed to assess the value created or destroyed by FMD and its control, and how this is distributed throughout society.

**Table 1:** Identified studies on the impact of FMD on smallholder systems. Existing studies have typically focussed on particular aspects of FMD impact

Country	Impact
Cambodia	Reduction in smallholder household income of 4.4–11.7% annually following an FMD outbreak. Loss of 54 – 92% of animal value following FMD infection (Shankar et al., 2012; Young et al., 2013). Effective biannual vaccination would be profitable even if outbreaks occurred only every 20 years without vaccination (Young et al., 2013)
	Most producers are subsistence farmers. A best practice invention involving improved husbandry and disease control (including FMD vaccination and biosecurity) more than doubled cattle daily weight gains (Young et al., 2014b) and income at least doubled for 53% of participants(Young et al., 2014a)
	Annual incidence during the 2010 outbreak was estimated to be about 13% for cattle and buffalo at US$247 per animal affected accounting for 10.6% loss of farm‐gate value of large ruminants. National vaccination control had an estimated benefit‐cost ratio of 1.40 (95% CI: 0.96–2.20) (Young et al., 2016)
Laos	Loss of 22–30% of animal value following FMD infection (Rast et al., 2010)
	FMD affected smallholder households experienced average losses of 16‐60% of household income depending on the region (Nampanya et al., 2015). FMD was estimated to cause national losses of >US$100 million in 2011 (Nampanya et al., 2016b). Impacts are felt for longer in poor villages (Nampanya et al., 2016a)
Philippines	In a largely backyard farming sector, FMD outbreaks caused pork and chicken wholesale prices to drop by about 15% affecting producers, traders, processors and retailers (Abao et al., 2014)
South Sudan	Loss of US$25 per cow per year in a region where 90% of the population have an income of <1 dollar a day (Barasa et al., 2008)
Pakistan	Reduction on milk yield in cattle and buffalo after infection. Milk yield only returned to two‐thirds the level of pre‐infection after 60 days (Ferrari et al., 2014)
Uganda	On farms that experienced outbreaks, costs per animal were far greater in smaller farms (US$123 versus US$17 on large farms), partly due to a lack of funds for vaccination and smallholders being compelled to sell stock at salvage prices due to lack of an alternative income (Baluka et al., 2014) Outbreaks halved the value of cattle and reduced cash crop production. If vaccination was effective if would pay for itself more than twice over (Rutadwenda, 2003)
Ethiopia	Many cattle were kept for draft power to cover for FMD affected cattle. Impacts largely occurred as reduced household food production and farmer welfare and not income due to limited market participation (Jemberu et al., 2014). Outbreaks in commercial dairy farms caused losses of almost US$2000 (Ashenafi, 2012). Milk constituted half of the daily diet of Borena pastoralists. About a quarter of cattle were infected within the last year or two. Infected cattle experienced milk reductions of >70% for about 1 month on average (Bayissa et al., 2011)
Botswana	Revenues from FMD‐free EU market access were absorbed by an inefficient system and not passed on to farmers. Access of small producers to export markets should be increased (through transport, government assistance, alternatives to fenced FMD‐free zones) (Botswana parliamentary inquiry, 2013)
Kenya	Closure of a large livestock market has a large effect on the peri‐unban poor, as 65% of the town (Garissa) depended upon the market for their livelihood (Yusuf, 2008). Large dairy farms in Kenya employ large numbers of poor workers with milking in large herds often done by hand. An outbreak in a large herd causes losses of US$15 000 (Mulei et al., 2001) to >US$100 000 per farm (Kimani et al., 2005)
Zimbabwe	Although 16% of the value of FMD‐free trade filters down to low‐income households, FMD control was of limited benefit to the poor who are more affected by other livestock ailments and poor husbandry, and are more dependent on poultry and goats than cattle. However, FMD has a large overall impact on the economies of Southern African countries (Perry et al., 2003)
Namibia	A cost benefit analysis of different FMD management options in endemic wildlife rich areas suggested that FMD control would have a positive but uncertain impact on poverty and a marginal benefit to smallholders through increased market access, with limited improvements in productivity (Cassidy et al., 2013)
Bolivia, Peru, Ecuador	FMD impact on smallholders differs even within the same area. While some producers are mainly affected financially during an outbreak, for others the impact is primarily on provision of household food affecting food security. The indirect impact for producers depends largely on the price paid for vaccine (which depends on the level of subsidization) and the number of susceptible animals owned by the household. Market closures have less effect on those living far from markets. Nationally, however, the cost of vaccination (including distribution and implementation) is the main impact of FMD in the three countries studied, reflecting the low incidence at this stage of the eradication campaign (Limón et al., 2014)
Tanzania	Milk losses affected cattle and goats, with two‐thirds of households in a randomized survey losing the capacity to sell milk as a result of FMD outbreaks in the last year. The same proportion were affected by loss of livestock traction due to FMD induced lameness (Casey et al., 2014). FMD was the most important disease for agro‐pastoralists, impacting both livestock and crop production

In terms of robust quantitative studies the literature is possibly stronger in Asia than Africa. Country circumstances may have changed since some studies were performed e.g. Zimbabwe in Perry et al. (2003). However, many of the findings have wider relevance, not restricted to a particular country at the time of the study.

An important over‐simplification of many studies predicting the benefits of control is a failure to incorporate the variable effectiveness of FMD control programmes (Knight‐Jones et al., 2014a, 2016; Knight‐Jones et al., 2015a, Lyons et al., 2015c; Elnekave et al., 2013; Woolhouse et al., 1996; Lyons et al., 2014). Two critical factors are (i) the variable potency and quality of vaccines used in endemic settings (Metwally et al., In press) and (ii) the limited application of biosecurity and sanitary control measures (Young et al., 2015).

Although useful research has been performed, the literature largely consists of small and unrelated pieces of work. Collectively they provide an incomplete picture that is dominated by knowledge gaps. The scarcity of FMD impact studies results in an inconclusive body of evidence, and the wider significance of findings remains disputed.

#### Household impact of an outbreak

In endemic countries, direct, visible FMD production losses vary and have been measured in different ways. Both large and small pig farmers and cattle holdings producing milk are typically the worst affected. This affects national output; in Kenya smallholders account for 70% of milk production (FAO, 2011).

In South Sudan annual losses resulting from reduced milk production and mortality from FMD were estimated at US$25 per head of cattle in the population (Barasa et al., 2008). In Pakistan outbreak milk losses over 60 days were put at US$100 per affected lactating cow (Ferrari et al., 2014). In Turkey estimated direct costs varied from US$152 per affected dairy heifer to US$294 for an affected lactating dairy cow, and about US$200 per affected animal for beef cattle (Şentürk et al., 2008).

Household impact can be more meaningful when put as percentage of income. In Cambodia smallholders experiencing outbreaks had household losses of about US$45, with low income households losing the largest percentage of income (12% of annual income for the poorest) (Shankar et al., 2012). Another study in Cambodia highlighted that impact per affected animal varied according to the animal's role and disease outcome, averaging US$216 for weight loss and treatment and US$371 if the animal was treated but died and replacement draught power was hired (Young et al., 2013). In Ethiopia estimated losses were US$137 per lactating cow in an outbreak and US$2175 per affected herd (Beyi, 2012). Jemberu et al. (2014) found that herd level impact of an outbreak was US$76 for Ethiopian crop‐livestock farmers, however this still constituted about 7% of annual income with 10% considered a catastrophic loss (Shankar et al., 2012). Even within this Ethiopian study, milk losses from individual cattle ranged from US$0 to US$176, depending on yield, duration of illness and severity of milk reduction.

Young et al. (2013) proposed that smallholders in Cambodia did not vaccinate against FMD, despite their study estimating that it would be profitable to do so, as farmers had a poor understanding of the benefits of vaccination, lacked the funds to purchase vaccine and did not appreciate the full cost of disease. One likely disincentive is the cost of vaccination as a proportion of household income, even if farmers perceived it as beneficial.

Many impact studies describe only financial impact on households with FMD cases. It is also useful to know the total population burden and the average household burden, showing which groups experience the greatest impact, considering all households at risk.

Long‐term follow‐up is required. A study of dairy cattle in Kenya found that after an outbreak, as well as poor fertility and milk yield, subsequent risk of mastitis was three times greater if a cow was clinically affected (Lyons et al., 2015a,b).

Impact varies according to intensity of production. Extensive smallholder cattle are often only traded when cash is required, so short‐term weight loss is less of a problem. This contrasts with more commercial systems where delays in the time taken for animals to reach finishing weights reduce profits. So in herds where productivity and efficiency are high, the impact of an FMD outbreak is great, but where productivity is already low FMD has a less dramatic impact, nonetheless, long‐term burden, although harder to measure, may still be significant, particularly if animals are chronically affected (Bayissa et al., 2011). Those highly dependent upon cattle milk for nutrition, including pastoralists and agro‐pastoralists, may experience reduced food security as a result of FMD, particularly affecting child nutrition (Barasa et al., 2008; Bayissa et al., 2011).

### Gaps in knowledge

#### Geographical ‐ regional and national

There is limited available information from West, Central and North Africa, India or China. India and China contain well over half of all the FMD susceptible livestock in virus‐endemic regions and have agricultural systems that are still heavily dependent on smallholder production (Knight‐Jones and Rushton, 2013).

#### Production systems

The main focus of the studies published is in milk systems where drops in output are easiest to measure. Little information is available from pastoral systems or meat producing systems. There is also a failure to consider the significant, but difficult to capture, invisible effects, such as change in herd structure and limiting the use of improved breeds, as well as indirect effects, such as restricted market and grazing access.

#### Species

Identified studies focused almost entirely on the impacts on cattle. This is natural given that cattle are the most susceptible to the disease and represent the major proportion of susceptible livestock biomass and value. However, small ruminants and pigs are very important in smallholder systems throughout regions where FMD is endemic, particularly for poorer livestock keepers that cannot afford cattle.

### Disease burden

#### Modelled

Using a simple mathematical model of FMD impact Knight‐Jones and Rushton (2013) extrapolated estimates of incidence, cost per case, vaccine usage and vaccine costs to derive an estimated annual global economic impact for FMD in endemic countries, considering only production losses and vaccination, of between US$6.5 and 21 billion. This burden falls mainly on smallholders and governments, with the highest incidence in cattle.

#### Serology

Numerous FMD sero‐prevalence surveys have been conducted in endemic countries. These studies reveal a high incidence of infection (Table 2). Sero‐positivity reflects prior infection with any serotype within the last 2 years or more (Elnekave et al., 2015). These surveys typically sample cattle 6–24 months old, therefore, sero‐positivity will reflect infection at some point in their life. However, the exact period of time at risk of infection is unknown, making it difficult to infer an exact incidence rate. Also, as animals acquire immunity over time, incidence will be lower in older cattle.

**Table 2:** Results from FMD sero‐prevalence surveys conducted in endemic countries where smallholder farming is widespread (McLaws et al., 2014). Sampling methodology will affect results (age, vaccination, strictly random‐versus targeted versus haphazard)

Country	Species	Year^a^	Study sero‐prevalence (range)	Sample size (animals)	Number of surveys^a^	Source	Study area
Botswana	SR	2006^a^	9	535	1	Hyera et al. (2006)	Provincial
Chad	LR	2009	36	796	1	Report	National
Egypt	LR	2011	19	2349	1	Report	National
Egypt	SR	2011	11	1144	1	Report	National
Ethiopia	LR	2006‐10	14%^b^ (8‐24)	46 831^‡^	9	See below^c^	Provincial+National (Ayelet)
India	LR	2010‐14	27%^b^ (26‐29)	193 845^‡^	5	Reports	National
India	SR	2009‐14	21%^b^ (12‐21)	18 189^‡^	5	Rout et al. (2014), Ranabijuli et al. (2010), Reports	National+Provincial (Ranabijuli)
Iran	LR	2011	54	8349	1	Emami et al. (2015), Report	Provincial
Jordan	SR	2007	8%^b^ (6‐10)	620^‡^	2	Al‐Majali et al. (2010)	Provincial
Kenya	LR	2008‐10	49%^b^ (45‐53)	4208^‡^	2	Kibore et al. (2013), Chepkwony et al. (2012)	Provincial+Almost national (Kibore)
Laos	LR	2005	36	5494	1	Blacksell et al. (2008)	National
Nigeria	LR	2009‐11	73	369	1	Lazarus et al. (2012)	Provincial
Pakistan	LR	2012^a^‐12	43%^b^ (19‐67%)	5400^‡^	2	Nawaz et al. (2014), Akram and Khan (2012)	Provincial
Pakistan	SR	2014^a^	21	1200	1	Ur‐Rehman et al. (2014)	Provincial
Rwanda	LR	2009	41	278	1	Uwizeye et al. (2009)	Provincial
Somalia	LR	2006‐9	35%^b^ (15‐58)	11 827^‡^	3	Medina (2010), Reports	Provincial+National (Medina)
Sudan	LR	2006‐8	79	469	1	Habiela et al. (2010)	National
Sudan	SR	2006‐8	24	403	1	Habiela et al. (2010)	National
Tanzania	LR	2014^a^	76	330	1	Mkama et al. (2014)	Wildlife interface
Turkey	LR	2009‐12	13%^b^ (10‐17)	95 112^‡^	3	Reports	National
Turkey	SR	2010‐12	20%^b^ (16‐24)	62 673^‡^	2	Reports	National
Uganda	LR	2007	39	309	1	Mwiine et al. (2010)	Provincial
Zimbabwe	LR	2009	18	228	1	Jori et al. (2016)	Wildlife interface

LR, Large ruminants (cattle, water buffalo); SR, Small ruminants (sheep and goats)

a For some surveys only year of reporting, not sampling,was known.

b For some countries the weighted average sero‐prevalence from >1 survey is given with the sum of the number of animals sampled from all surveys combined.

c Bayissa et al. (2011), Molla et al. (2010), Megersa et al. (2009), Gelaye et al. (2009), Alemayehu et al. (2014), Mekonen et al. (2011), Yahya et al. (2013), Ayelet et al. (2012), Mohamoud et al. (2011).

Antibodies against non‐structural proteins are typically assessed. These antibodies are produced after infection with FMD virus and after FMD vaccination unless a purified vaccine was used. Few of the countries included in Table 2 use purified vaccines (Turkey does), so the proportion sero‐positive may include vaccinated animals that have not been infected. However, vaccination levels are typically low in cattle and negligible in small ruminants in most (but not all) of these countries.

Impact relates to the level of disease. Unfortunately the proportion of sero‐positive animals that show clinical disease is rarely reported and remains uncertain, varying with factors such as breed, age, maternal immunity, vaccination status and virus strain. In outbreak investigations of FMD serotype Asia‐1 in mostly vaccinated cattle in Turkey, clinical disease was detected in 72% of cattle with serological evidence of infection (Knight‐Jones et al., 2014a). Yet in a heavily vaccinated dairy farm in Israel no clinical cases were detected in some small groups with low sero‐prevalence (Elnekave et al., 2013). So although the sero‐prevalence surveys reported in Table 2 suggest that about one in three young cattle become infected with FMD virus, the proportion that develop disease, and disease severity, is uncertain. This uncertainty is an important limitation when inferring impact.

#### Observational studies

Foot‐and‐mouth disease outbreaks are typically under‐reported in endemic countries. From the few cohort studies, or studies assessing under‐reporting that have been conducted in endemic countries, a high FMD incidence is not unusual (Casey et al., 2014; McLaws, 2012; Bronsvoort et al., 2003).

In a study in Cameroon just over half the herds experienced FMD per year. Traditional extensive livestock systems often rely on local and distant communal grazing. Herds directly contact several other herds every day, leading to high levels of disease transmission (Bronsvoort et al., 2003). Herds that are sedentary and do not use communal grazing have a lower risk (Knight‐Jones et al., 2014a). The expectation is that incidence will be lower when stocking densities are low and there is less contact between animals and groups, as there are fewer opportunities for transmission.

In a cross‐sectional survey in rural Tanzania, >80% of agro‐pastoralists (those with smallholdings that also use extensive grazing) and pastoralists experienced FMD outbreaks in the year assessed, with each outbreak affecting half of all cattle (71% of adult female cattle) and a third of goats. For sedentary smallholders about a third experienced outbreaks within the last year (Casey et al., 2014). Whether these findings represent similar production systems elsewhere is not certain, however, they do show that many pastoralist and sedentary smallholders in large areas of East and West Africa experienced an extremely high FMD incidence.

#### Ranking

The importance of FMD varies by production type. In the same Tanzanian study FMD was ranked as the most important livestock disease by agro‐pastoralists; for pastoralists FMD was the second most important, after East Coast Fever (ECF). For sedentary smallholders, FMD was ranked third after ECF and anthrax/black‐leg. This mirrored lower sero‐prevalence in the latter group (although still ~40%) (Casey et al., 2014). A study of pastoralists in Kenya found FMD was again ranked as being the highest impact livestock disease after ECF (Onono et al., 2013). Pastoralists in Borena, Ethiopia reported that FMD was the most important cattle disease (Jibat et al., 2013).

A WorldBank survey of African governments reported FMD as the livestock disease with the third biggest impact on poverty after ectoparasitosis and Newcastle's disease (Le Gall and Leboucq, 2004). However, in a different survey of national veterinary services in Africa, only 11% of countries listed FMD as the highest priority disease for smallholders. However, FMD was listed as an overall priority by more countries than any other livestock disease, including zoonoses, although the position of FMD on the priority list varied (Grace et al., 2015). Government priorities do not always reflect smallholder priorities. A study of smallholders in Zimbabwe found that whereas the government prioritized FMD control, smallholders prioritized parasitic diseases (Chatikobo et al., 2013).

The above findings are reflected in a review of prioritization studies by Perry and Grace (2009) which found that FMD was consistently prioritized, including by studies focussing on poverty reduction, however, there was variation in its exact rank (Perry and Grace, 2009).

### Overall findings

Bias may sometimes be an issue, as FMD studies are likely to be performed in areas where FMD is important and control is being considered. However, a simple but logical conclusion would be that, although uncertain, population level impact will be high in clusters where FMD is prevalent, particularly for those dependent on commodities affected by FMD, such as milk (pastoralists and commercial dairy farmers) or pigs, and where national economies depend on access to FMD‐free export markets. Hence, some sub‐groups will experience a high impact while others do not, even within the same country.

## FMD smallholder impact: how can we find out more?

### Measuring the impact of disease and control

#### Framework of FMD impact

To help guide future investigations we have categorized and summarized the information needed for FMD impact studies in a framework (Table 3). This framework considers what is known about these different categories of impact information and their likely significance. Various data sources have to be utilized and some parameters may have to be modelled using *ad hoc* approaches to overcome limitations in available data (Young et al., 2016).

**Table 3:** Framework of FMD impacts, considering their significance, the extent of our knowledge and ease of estimation for each impact

Impacts	Significance/Knowledge/Ease	Gaps
*Visible production losses*		
Milk losses – short‐term and long‐term	Significance – High Knowledge – Limited Ease of estimation – Moderate but variable	Some studies have estimated short to medium term losses. Losses over a cow's lifetime will be significantly greater Easier to measure in some smallholder dairies, but difficult to measure in pastoral systems or when calves are suckling. Uncertain affect on milk quality and how losses translate into reduced nutrition and food security
Loss of draught power	Significance – Variable Knowledge – Limited Ease of estimation – Difficult	Has been considered but is hard to quantify due to the seasonality of demand for animal power. Lameness may contribute to other production losses, e.g. through reduced grazing and water access, and fertility
Reduced weight gains, poor feed conversion	Significance – High Knowledge – Limited Ease of estimation ‐ Moderate	Some studies have estimated short to medium term losses. Losses over a cow's lifetime may be significantly greater
Deaths	Significance – Moderate Knowledge – Limited Ease of estimation – Simple	Few descriptions of outbreaks accurately describe mortality. Estimates are often based on opinion and reported/unconfirmed cases
*Invisible production losses*		
Reduced fertility	Significance – High Knowledge – Limited Ease of estimation ‐ Moderate	As a long‐term impact this has not been captured but could be modelled
Changes in herd structure	Significance – Variable Knowledge – Limited Ease of estimation – Difficult	As a consequence of reduced fertility more adults will be maintained per unit of outputs (milk, cattle for meat) leading to an overall need for greater feed inputs per unit of output
Delay in the sale of animals and products	Significance – Variable Knowledge – Limited Ease of estimation – Difficult	Timing of sales may be suboptimal as a consequence of reduced weight gains or salvaging cull animals
*Expenditure – additional costs*		
Vaccines	Significance – High Knowledge – Adequate Ease of estimation – Simple	Variable but easy to measure
Vaccine delivery/administration	Significance – High Knowledge – Adequate Ease of estimation – Moderate	Will vary depending on the setting but can be measured
Movement control	Significance – High Knowledge – Limited Ease of estimation – Difficult	Despite its importance the impact of movement controls is complex and seldom measured
Surveillance and diagnostic tests	Significance – Moderate Knowledge – Adequate Ease of estimation – Simple	Rarely quantified
Culled animals	Significance – High Knowledge – Limited Ease of estimation – Moderate	Direct culling of FMD affected animals can easily be estimated, but culling at a later stage for low productivity resulting from FMD is harder to measure
*Reactions leading to revenue forgone*		
Use of suboptimal breeds and production systems	Significance – High Knowledge – Limited Ease of estimation – Difficult	FMD may be one of many factors contributing to this
Denied access to markets	Significance – High Knowledge – Limited Ease of estimation – Difficult	Includes not only international trade in FMD‐free markets but also trade between endemic countries and domestic trade, the latter are particularly hard to estimate. Trade barriers other than FMD may also prevent trade.

For endemic settings there is a need to consider the current impact of FMD, followed by an assessment of possible control measures, their effectiveness and cost. A benefit‐cost‐analysis can then assess impact over time when control measures are in place, examining who benefits and who pays, either in terms of monetary investments or diversion of resources, including time utilized for FMD control. Benefits and costs will vary for different groups during the long course of a control programme.

#### Approaches and considerations

A discussion of possible study designs is included in the Supplementary Material (ESM Appendix S1). Ideally the analysis would be layered between farm‐level assessments, sector level assessments and the whole economy. Such a structured approach allows the identification of the winners and losers from a control process.

1. Herd level impacts
  - This requires modelling the dynamics of the herd to see how herd structure, productivity and efficiency are impacted by FMD.
2. Farm–household level impacts
  - Impact on household resource allocations where families are involved in multiple activities could be captured in a linear programming approach.
  - A simple description of the impact experienced by households during outbreaks is needed, detailing who is affected (men, women, children) and how (less food, more work, more stress).
3. Sector and economy wide impacts
  - FMD reduces flows of animals and products to the market and this could be captured in economy surplus models in simple economies where the food system is relatively direct.
  - In more complex economies there could be a need for sector and possibly economy wide models.
  - Assessments would have to capture how benefits and costs affect different groups (public sector and revenues, holdings of different types and location including those outside FMD‐free zones, consumers, environment and wildlife).

#### Ex ante and ex post

*Ex ante* analysis requires modelling methods and parameterization based on previous outbreaks. Predictions from *ex ante* studies will be uncertain, however, results can be useful so long as assumptions and methods are clear, and understood. *Ex post* analysis requires detailed field studies with collection of data from affected regions, considering all dimensions including animal, herd, household and the economy (Shankar et al., 2012). Outputs should illustrate the impacts across gender, age and class groups. Very few attempts have been made to capture changes *ex post* for any disease outbreak (Rushton and Gilbert, 2014) and more *ex post* studies should be performed, preferably using consistent approaches to allow different studies to be combined or compared.

### Wider impacts and complexities of control

#### Challenges when assessing impact and control

Foot‐and‐mouth disease impacts on the wider economy affecting those that do not keep livestock. The disease, and its control, influence the demand for certain goods and services, and alter the supply and prices of finished livestock and meat (Abao et al., 2014). These sector level impacts have been captured by assessments of outbreaks in FMD‐free countries (Buetre et al., 2013), yet are rarely described in the endemic setting. Furthermore, some FMD control measures may also reduce the impact of other diseases.

Impact on food security is hard to assess and could be mediated through several different impacts of FMD, including losses in milk production, body condition, mortality and traction power (Casey et al., 2014; Barasa et al., 2008; Nampanya et al., 2016a; Jemberu et al., 2014). If losses reduce the amount and variety of food available for home consumption, and these losses are not replaced with purchased food, nutritional shortages are likely to arise. However, little is known about the vulnerability of affected households, which will depend on the availability of alternative sources of food and income.

National policy should consider population level impact. Mortalities are relatively easy to capture and high mortality rate diseases are often prioritized above common, low mortality rate diseases such as FMD or parasitism. However, low mortality diseases may still have a high impact due to high incidence. That said, the impact of low mortality diseases is reduced if herd size is more important than productivity and profitability. This is common for farmers in extensive settings in developing countries, where in addition to being a measure of socio‐economic status, herd size may be used as both a bankable asset, with cattle sold when cash is needed, and as a strategy for increasing the chance of herd survival during mass mortality events.

As FMD eradication is not foreseeable for many endemic countries, an assessment of whether control is cost‐effective if it achieves a reduced incidence with ongoing mass vaccination is required. Control costs may remain high even when incidence is low, as population level control measures are still required. The level of incidence reduction required for positive returns should be determined. Findings will be specific for a given setting.

#### Externalities and who should pay

If some farmers do not control FMD, other farmers will also continue to experience a raised disease risk. This undermines control efforts and may discourage individuals from investing in FMD control. Conversely, private investment in FMD control by large enterprises may reduce FMD risk for smallholders. These externalities must be considered as they will influence the efficacy of the population level response needed to control FMD (Knight‐Jones and Rushton, 2013).

Where externalities arise, the state needs to consider how to reallocate these gains and costs through taxation and disease control support. The state may restructure markets through subsidies or levies, applied to inputs or outputs, in order to favour those that benefit the least, or suffer the most, from FMD control. This would require complex judgements on how to achieve this equitably. The balance of public and private investment in disease control is always important, yet little is known about farmers’ willingness to pay for FMD control.

#### International, regional and local trade

The impact of FMD on trade is particularly important for those able to export beef (Knight‐Jones and Rushton, 2013). However, in addition to FMD there may be multiple reasons why a country cannot access lucrative export markets, e.g. inability to produce beef of reliable quality and quantity at a competitive price, or difficulties complying with other sanitary standards (Rich et al., 2009). Remaining trade barriers will reduce the benefits of FMD control. Furthermore, export trade requirements may be dictated by powerful trade partners who demand increasingly restrictive and costly control measures. Market access can be fragile as outbreaks are unpredictable and countries, including developed countries, may apply trade bans without regard for accepted international trade standards. Sometimes these bans are illogical, arbitrary and opportunistic.

Foot‐and‐mouth disease status is sometimes used to block exports from one endemic country to another. Over 4 million livestock are exported from the Horn of Africa to the Middle East each year (FEWS‐NET, 2010). Many poor farmers are directly affected when shipments are rejected because of FMD sero‐positivity despite both regions being FMD‐endemic. According to World Trade Organisation standards, importing countries should not block trade because of the presence of a notifiable disease in the exporting country if the disease is also present in the importing country. That said, important strain and serotype differences do exist between East Africa and the Middle East, and incursions of novel strains could have a massive impact on the wider region and beyond (Knight‐Jones et al., 2014b).

Local markets in endemic countries also close during FMD outbreaks (Yusuf, 2008). Furthermore, FMD can affect many commodities; outbreaks in Zambia in 2010 resulted in Botswana banning the import of Zambian maize bran (Sinkala et al., 2014).

It is worth noting that despite a high FMD burden (Ganesh Kumar, 2012), a large smallholder sector and FMD related export bans, India exported more beef by volume than any other country in the world in 2014 (USDA/FAS, 2015). This suggests that, when competitively priced, beef exports can thrive without freedom from FMD. However, India's beef exports may fall; China, which has banned Indian beef because of FMD, may clamp down on Indian beef coming via Vietnam, India's biggest market (India Times, 2015), and Russia has recently applied FMD related trade bans (ProMED, 2015).

#### Equality and poverty alleviation

Whilst wealthy farms with export potential benefit from elevated national FMD status (Scoones et al., 2010), 16% of the benefits of the FMD‐free beef export trade were estimated to filter down to low‐income households (Perry et al., 2003). However, this is a small proportion if the export sector is developed specifically to alleviate poverty. Smallholders in South America have benefited from the beef export trade by supplying stock for larger farms more directly involved in exports. In Botswana and Namibia beef from smallholder cattle can go to higher priced export markets that only accept beef from countries or zones that are free from FMD. However, additional barriers exist as complying with other sanitary standards, including traceability and pre‐slaughter residency requirements, is challenging, particularly for animals from extensive smallholdings (Knight‐Jones, 2015).

Within a country, movement restrictions and zonation disrupt domestic trade and access to rangelands. Farmers outside FMD‐free zones, which are typically dominated by smallholders, do not have access to markets available to those within the FMD‐free zones, yet they may still be subject to the burden of control measures. Even within FMD‐free zones, the benefits of control are distributed unevenly (Barnes, 2013; Cassidy et al., 2013; Randolph et al., 2005).

#### Ecological impact

Traditional approaches to control in regions of Africa where wildlife carrying FMD virus are present have often incorporated the use of cordon fences to separate wildlife populations in which FMD viruses are endemic from FMD‐free livestock. This largely concerns African Buffalo (*Syncerus caffer*) which often carry SAT serotype viruses without showing clinical disease. This restriction of movement has a severe impact on wildlife populations and the environment, with knock on effects on wildlife tourism (Ferguson et al., 2013).

## Feasibility of control

### FMD control options in smallholder systems

If control is unsuccessful the negative impacts of control will be experienced without obtaining the benefits. Irrespective of the impact of the disease, control should not be attempted unless effective control is feasible. Movement restrictions are extremely difficult to implement in smallholder settings in poor countries as smallholders often require continued access to local markets and communal grazing. In much of Africa land is owned by the state and farmed communally, under these circumstances a farmer can do little to prevent virus exposure if other livestock at shared grazing are infected.

Disease control in these settings may be heavily reliant upon vaccination alone. But FMD vaccines used in endemic countries typically provide short‐lived and limited protection (Knight‐Jones et al., 2015). Furthermore, mass vaccination of all cattle and pigs, and possibly sheep and goats, every 6 months, may be unrealistic in developing countries with poor infrastructure and numerous smallholdings (Knight‐Jones et al., 2016). Whether or not FMD can be controlled in endemic countries using quality, high potency vaccines, with minimal movement restrictions, remains an important, yet unanswered question.

Although FMD can be controlled in smallholder systems, in parts of Southern Africa the presence of endemically infected wildlife makes control more challenging. In addition, although the effectiveness of vaccination programmes is an issue in many FMD‐endemic countries, this is particularly so for SAT‐2 strains that circulate in Southern Africa (Thomson et al., 2017; Bastos et al., 2003; Doel, 2003; Bari et al., 2014; Knight‐Jones et al., 2016). Where zonal FMD‐freedom has been achieved in Southern Africa, an ongoing threat of new outbreaks results in burdensome restrictions and periodic epidemics with suspension of beef exports (Barnes, 2013; Cassidy et al., 2013).

### Wildlife and smallholder friendly approaches to FMD control

Sanitary trade standards exist to ensure the status of the final product exported. The risk of FMD virus surviving in appropriately matured, de‐boned beef is very low if not negligible (Paton et al., 2010). This is recognized in international standards (OIE, 2015), which support the export of these commodities to countries that are free from FMD, even if the beef was produced in FMD‐endemic regions (Thomson et al., 2009, 2013b); an approach referred to as commodity‐based‐trade. This provides an approved way for smallholders to access lucrative beef export markets without the need for control measures that have excessive negative impacts on producers, wildlife and the environment.

This approach has been advocated for Southern Africa where beef is produced in areas with FMD virus infected wildlife (Thomson et al., 2013a). Such regions may benefit from recent OIE code changes that permit the export of deboned‐beef from healthy, vaccinated cattle in endemic zones where an official FMD control programme exists, without requiring local geographic proof of FMD‐freedom as long as the animal passes through a quarantine station before slaughter (OIE, 2015).

## Conclusions

Relatively little has been done to assess FMD impact on smallholders, particularly in Africa. The literature is patchy but shows that FMD impact is high for some smallholders but low for others. Impact will vary with disease incidence, level of dependency on aspects of production most affected by FMD and the positive, and negative, impact of control measures. FMD affects the efficiency of production by reducing milk yields, livestock growth rates and fertility, and restricting the use of productive breeds that are highly susceptible to FMD. Control costs arise from vaccination, movement restrictions, wildlife controls, restricted market access and culling.

A key impact that is difficult to measure is the failure to achieve efficient production due to the threat of FMD even when livestock are healthy. FMD discourages investment to increase productivity and smallholders in FMD‐endemic regions often adopt low input–low output approaches that are more resilient to FMD. However, there are many other reasons why more productive and efficient approaches are not adopted by smallholders, including limited access to capital and markets, other diseases, particularly endo‐ and ecto‐parasites, and inadequate technical knowledge, infrastructure and support.

The global average impact of FMD on smallholders is not known, however, this would be a fairly meaningless statistic as it encompasses such a heterogeneous group and impact is to some extent specific for a given setting. Studies have identified instances when impact is high and further defining the characteristics of smallholders most affected by FMD would be useful. The costs and benefits of control are also situation specific, and are unequally distributed between different groups within the livestock sector and the wider national economy. This is complex and requires further investigation.

Fundamental components of FMD control, such as vaccination, biosecurity and livestock movement controls, are often inadequately implemented in smallholder systems in developing countries. In some regions infected wildlife present an additional challenge for those attempting FMD eradication, although the livestock disease burden attributable to wildlife transmission is disputed. Before investing in control measures, consideration must be given to their likely impact, including negative impacts. Although mortality from FMD is low, high disease incidence can lead to a heavy burden at the population level, however, controlling FMD in smallholder systems in developing countries is costly, challenging and requires long‐term commitment.

## Supporting information


**Appendix S1**. Challenges when assessing the impact of FMD control ‐ Study designs
**Appendix S2**. Review bibliography
**Table S1.** Experts contacted to obtain published and unpublished work on the impact of FMD on smallholder farmers.
