# Supplementary material for: Foot‐and‐Mouth Disease Impact on Smallholders ‐ What Do We Know, What Don't We Know and How Can We Find Out More?
Source: Transbound Emerg Dis. 2016 May 11;64(4):1079–94. doi: 10.1111/tbed.12507 (PMC5516236; doi:10.1111/tbed.12507)
Supplement: Supplementary file 1 — Appendix S1. Challenges when assessing the impact of FMD control ‐ Study designs Appendix S2. Review bibliography Table S1. Experts contacted to obtain published and unpublished work on the impact of FMD on smallholder farmers. [file TBED-64-1079-s001.pdf]

Electronic Supplementary Material

Foot-and-mouth disease impact on smallholders - What do we know, what don't we know and how can we find out more?

T. J. D. Knight-Jones and J. Rushton

Appendix S1 - Supplementary Text

Challenges when assessing the impact of FMD control

Study designs

With such a complex disease with so many unknowns it may appear to be simplest to compare either a) locations that are similar except for differing levels of FMD, b) the same region before and after a change in FMD burden or c) to avoid confounding, conduct a cluster randomised trial to estimate the economic benefits of control in otherwise similar regions. Another option would be to model the effect of control considering the most important factors only, to avoid over-complexity. However, a large degree of speculation and uncertainty is unavoidable as many aspects that would need to be predicted are highly unpredictable.

Another option would be to extrapolate from studies that measure the impact of outbreaks at farm or village level. This could be done using national farm demographic and FMD incidence data. Such studies would require information on farm and animal production from prior, during and after an outbreak. Although less relevant for smallholdings, in some countries there are sufficient animals on herd health monitoring schemes that can be utilised (Lyons et al., 2015c,

Lyons et al., 2015a, Lyons et al., 2015b). In other settings there would be a need to establish monitoring systems or use records of sales. In addition to the production losses, there is a need to collect information on the costs of surveillance, control and prevention. These are normally available for the public sector, yet are rarely captured properly at the farm level. Again a systematic process of data collection would be helpful, either through surveys or measures of pharmaceutical usage.

Accounting for trade and movement impacts remains uncertain as the current economic models often fail to capture how an economy absorbs a shock and rebounds.

References

Lyons, N. A., N. Alexander, K. D. Stärk, T. D. Dulu, J. Rushton and P. E. Fine, 2015a: Impact of foot-and-mouth disease on mastitis and culling on a large-scale dairy farm in Kenya. *Vet Res,* 46, 41.

Lyons, N. A., N. Alexander, K. D. Stärk, T. D. Dulu, K. J. Sumption, A. D. James, J. Rushton and P. E. Fine, 2015b: Impact of foot-and-mouth disease on milk production on a large-scale dairy farm in Kenya. *Prev Vet Med*.

Lyons, N. A., K. D. Stärk, C. van Maanen, S. L. Thomas, E. C. Chepkwony, A. K. Sangula, T. D. Dulu and P. E. Fine, 2015c: Epidemiological analysis of an outbreak of foot-and-mouth disease (serotype SAT2) on a large dairy farm in Kenya using regular vaccination. *Acta Trop,* 143, 103-111.

Appendix S2 - Review bibliography

Papers identified and retained.

Conducted August 2014 - Further references are included in the main paper bibliography.

General

Garabed, R.B., Johnson, W.O., Gill, J., Perez, a.M., Thurmond, M.C., 2008. Exploration of associations between governance and economics and country level foot-and-mouth disease status by using Bayesian model averaging. Journal of the Royal Statistical Society: Series A (Statistics in Society) 171, 699-722.

James, A.D., Rushton, J., 2002. The economics of foot and mouth disease. Revue scientifique et technique (International Office of Epizootics) 21, 637-644.

Jarvis, L.S., Cancino, J.P., Bervejillo, J.E., 2005. The effect of foot and mouth disease on trade and prices in international beef markets - American Agricultural Economics Association annual meeting; Providence, Rhode Island, July 24-7 2005.

Junker, F., Komorowska, J., Tongeren, F.V., 2009. Impact of Animal Disease Outbreaks and Alternative Control Practices on Agricultural Markets and Trade: The case of FMD. OECD Food, Agriculture and Fisheries Working Papers, No. 19, OECD Publishing. 1-18.

Knight-Jones, T.J., Rushton, J., 2013b. The economic impacts of foot and mouth disease - what are they, how big are they and where do they occur? Prev Vet Med 112, 161-173.

Leon, A., Hoet, J., Gobernación, Z., Maracaibo, Z., Services, I., 2008. Estimated Direct Economic Losses Caused by a Severe Foot and Mouth Disease Outbreak in a Typical Farm in the State of Zulia. Profit, 3-3.

Perry, B.D., Randolph, T.F., 2003. The economics of foot-and-mouth disease, its control and its eradication. In: Foot-and-mouth disease: control strategies. In: Dodet, B., Vicari, M. (Eds.) Éditions scientifiques et médicales Elsevier SAS, 23-41.

Perry, B.D., Rich, K.M., 2007. Poverty impacts of foot-and-mouth disease and the poverty reduction implications of its control. Vet Rec 160, 238-241.

Perry, B.D., Sones, K.R., 2007. Global roadmap for improving the tools to control Foot-and-Mouth disease in endemic settings. Report of a workshop held at Agra, India 29 Novemebr-1 December 2006, and subsequent Roadmap outputs, ILRI (International Livestock Research Institute), Nairobi.

Rich, K.M., Perry, B.D., 2010. The economic and poverty impacts of animal diseases in developing countries: New roles, new demands for economics and epidemiology. Preventive Veterinary Medicine 101, 133-147.

Rushton, J., 2008. Economic aspects of foot and mouth disease in Bolivia. Rev Sci Tech 27, 759-769.

Rushton, J., Knight-Jones, T.J.D., 2012. The impact of foot and mouth disease. OIE-FAO report.

www.oie.int/doc/ged/D11888.PDF

Sumption, K., Rweyemamu, M., Wint, W., 2008. Incidence and distribution of foot-and-mouth disease in Asia, Africa and South America; combining expert opinion, official disease information and livestock populations to assist risk assessment. Transboundary and emerging diseases 55, 5-13.

Thomson, G.R., Penrith, M.L., Atkinson, M.W., Thalwitzer, S., Mancuso, A., Atkinson, S.J., Osofsky, S.A., 2013b. International trade standards for commodities and products derived from animals: the need for a system that integrates food safety and animal disease risk management. Transbound Emerg Dis 60, 507-515.

Thornton, P.K., Kruska, R.L., Henninger, N., Kristjanson, P.M., Reid, R.S., Atieno, F., Odero, A., Ndegwa, T., 2002. Mapping poverty and livestock in the developing world. International Livestock Research Institute, Nairobi, Kenya. 124 pp.

Africa

Aklilu, Y., Catley, A., 2010. An Analysis of Benefits by Wealth Group and Policy Implications Livestock Exports from Pastoralist Areas. Policy.

Ashenafi, B., 2012. Costs and benefits of foot and mouth disease vaccination practices in commercial dairy farms in Central Ethiopia. MSc thesis.

Barasa, M., Catley, A., Machuchu, D., Laqua, H., Puot, E., Tap Kot, D., Ikiror, D., 2008. Foot-and-Mouth Disease Vaccination in South Sudan: Benefit–Cost Analysis and Livelihoods Impact. Transboundary and emerging diseases 55, 339-351.

Bayissa, B., Ayelet, G., Kyule, M., Jibril, Y., Gelaye, E., 2011. Study on seroprevalence, risk factors, and economic impact of foot-and-mouth disease in Borena pastoral and agro-pastoral system, southern Ethiopia. Tropical Animal Health and Production 43, 759-766.

Botswana parliamentary inquiry, 2013. Final report of the special select committee of inquiry on the Botswana meat commission and the decline of the cattle industry. February - August 2013.

Cumming, D.H.M., Hargreaves, S., Rich, K.M., Catley, A., Cooke, M., Moyo, S., Donaldson, A., Strydom, P.J., 2010. Responses and reactions to Scoones et al . to ‘ Foot-and-mouth disease and market access : challenges for the beef industry in southern Africa ’. Pastoralism 1.

Ellis, P.R., Putt, S.N.H., 1981. The epidemiological and economic implications of the Foot and Mouth Disease vaccination programme in Kenya. Pan Livestock Services, 24 Albert Road, Caversham, Reading, RG4 7PE.

FEWS-NET, 2010. Cross-border Livestock Trade Assessment Impacts of lifting the livestock import ban on food security in Somalia, Ethiopia and the Djibouti borderland. Nairobi,Kenya.

Gadd, M.E., 2011. Barriers, the beef industry and unnatural selection: A review of the impact of veterinary fencing on mammals in Southern Africa - in Fencing for Conservation. In: Somers, M.J., Hayward, M. (Eds.) Springer, New York, USA, 153-186.

Hunter, P., 1998. Vaccination as a means of control of foot-and-mouth disease in sub-saharan Africa. Vaccine 16, 261-264.

Jabra, H.A., 2010. Foot and mouth disease surveillance in central and Southern Somalia. FAO/EuFMD Eastern Africa FMD Workshop, Narobi, Kenya, 8th - 12th February 2010.

Jibat, T., Admassu, B., Rufael, T., Baumann, M.P.O., Pötzsch, C.J., 2013. Impacts of foot-and-mouth disease on livelihoods in the Borena Plateau of Ethiopia. Pastoralism: Research, Policy and Practice 5.

Kimani Mwirigi, J.W., Murithi, R.M., 2005. Financial Impact Assessment of Foot and Mouth Disease in Large Scale Farms in Nakuru District, Kenya. Journal of the Kenya Veterinary Association 29, 7-9.

Mapitse, N., 2008. Botswana’s foot-and-mouth disease and beef trade policy. Steps-Centre.

http://steps-centre.org/wp-content/uploads/VetScience_Briefing_Botswana.pdf

Mazengia, H., Taye, M., Negussie, H., Alemu, S., Tassew, A., 2010. Incidence of foot and mouth disease and its effect on milk yield in dairy cattle at Andassa dairy farm, Northwest Ethiopia. Agriculture and Biology Journal of North America 1, 969-973.

Megersa, B., Beyene, B., Abunna, F., Regassa, A., Amenu, K., Rufael, T., 2009. Risk factors for foot and mouth disease seroprevalence in indigenous cattle in Southern Ethiopia: the effect of production system. Trop Anim Health Prod 41, 891-898.

Oarabile, L., 1994. The epidemiology and economics of foot-and-mouth disease in Botswana. MSc dissertation, VEERU (The Veterinary Epidemiology and Economics Research Unit), University of Reading, Reading, UK.

Onono, J.O., Wieland, B., Rushton, J., 2013. Constraints to cattle production in a semiarid pastoral system in Kenya. Trop Anim Health Prod.

Perry, B.D., Randolph, T.F., Ashley, S., Chimedza, R., Forman, A., Morrison, J., Poulton, C., Sibanda, L., Tebele, N., Stevens, C., Yngström, I., Forman, T., 2003a. The impact and poverty reduction implications of foot and mouth disease control in southern Africa. Proceedings of the

10th International Symposium on Veterinary Epidemiology and Economics, 2003. In, International Livestock Research Institute (ILRI), Nairobi, Kenya, 1-152.

Perry, B.D., Randolph, T.F., Ashley, S., Chimedza, R., Forman, T., Morrison, J., Poulton, C., Sibanda, L., Stevens, C., Tebele, N., Yngström, I., 2003b. The impact and poverty reduction implications of foot and mouth disease control in southern Africa, with special reference to Zimbabwe. International Livestock Research Institute (ILRI), Nairobi, Kenya, 152 pp. and CD-ROM.

Putt, S.N.H., Shaw, A.P.M., Woods, A.J., Tyler, L., James, A.D., 1987. Epidémiologie et économie vétérinaire en Afrique. In Manuel à l'usage des planificateurs de la santé animale. Veterinary Epidemiology and Economics Research Unit. Department of agriculture, University of Reading, Berkshire, UK.

Randolph, T.F., Morrison, J.A., Poulton, C., 2005. Evaluating equity impacts of animals disease control: The case of Foot and Mouth Disease in Zimbabwe. Review of Agricultural Economics 27, 465-472.

Rich, K.M., Perry, D., Kaitibie, S., 2009. Commodity-based Trade and Market Access for Developing Country Livestock Products : The Case of Beef Exports from Ethiopia. Int Food and Agribusiness Management Review 12, 1-22.

Rufael, T., Catley, A., Bogale, A., Sahle, M., Shiferaw, Y., 2008. Foot and mouth disease in the Borana pastoral system, southern Ethiopia and implications for livelihoods and international trade. Tropical Animal Health and Production 40, 29-38.

Scoones, I., Bishi, A., Mapitse, N., Moerane, R., Penrith, M.L., Sibanda, R., Thomson, G., Wolmer, W., 2010. Foot-and-mouth disease and market access: challenges for the beef industry in southern Africa. Development 1(2), 135-164.

Scoones, I., Wolmer, W., 2007. Land, Landscapes and Disease: The Case of Foot and Mouth in Southern Zimbabwe. South African Historical Journal 58, 42–64.

Thomson, G., 2008. A short overview of regional positions on foot-and-mouth disease control in southern Africa. Development.

Thomson, G.R., 1995. Overview of foot and mouth disease in southern Africa. Rev Sci Tech 14, 503-520.

Thomson, G.R., Penrith, M.L., Atkinson, M.W., Atkinson, S.J., Cassidy, D., Osofsky, S.A., 2013a. Balancing livestock production and wildlife conservation in and around southern Africa's transfrontier conservation areas. Transbound Emerg Dis 60, 492-506.

Thomson, G.R., Penrith, M.L., Atkinson, M.W., Thalwitzer, S., Mancuso, A., Atkinson, S.J., Osofsky, S.A., 2013b. International trade standards for commodities and products derived from animals: the need for a system that integrates food safety and animal disease risk management. Transbound Emerg Dis 60, 507-515.

Yusuf, M.M., 2008. The impact of quarantine restriction on livestock markets with special reference to foot and mouth disease in Garissa district, Kenya. MSc project.

Asia

Ellis, P.R., James, A.D., 1976. Foot and Mouth Disease: India. Conference proceedings: New Techniques in Veterinary Epidemiology and Economics, University of Reading, UK, 1976 (ISVEE 1). 118-122.

Kazimi, S.E., Karam, S., 1980. Effect on production performance in cattle due to Foot-and-Mouth Disease. Bulletin Office International d'Epizootis 92, 159-166.

Khounsy, S., Conlan, J.V., Gleeson, L.J., Westbury, H.A., Colling, A., Paton, D.J., Knowles, N.J., Ferris, N.P., Blacksell, S.D., 2008. Foot and mouth disease in the Lao People's Democratic Republic: I. A review of recent outbreaks and lessons for control programmes. Rev. sci. tech. Off. int. Epiz 27, 839-849.

Mannan, M., Siddique, M., Uddin, M., Parvaz, M., 2010. Prevalence of foot and mouth disease (FMD) in cattle at Meghna upazila in Comilla in Bangladesh. Journal of the Bangladesh Agricultural University 7, 317-319.

McLeod, R., 2011. Realised and Potential Economic Benefits of the Southeast Asia Foot and Mouth Disease Campaign. Development.

Nampanya, S., Khounsey, S., Abila, R., Dy, C., Windsor, P.A., 2014a. Household financial status and gender perspectives in determining the financial impact of Foot and Mouth Disease in Lao PDR. Unpublished.

Nampanya, S., Khounsy, S., Phonvisay, A., Young, J.R., Bush, R.D., Windsor, P.A., 2013. Financial Impact of Foot and Mouth Disease on Large Ruminant Smallholder Farmers in the Greater Mekong Subregion. Transbound Emerg Dis.

Nampanya, S., S. Khounsy, R. Abila, J. R. Young, R. D. Bush and P. A. Windsor, 2015: Financial Impacts of Foot-and-Mouth Disease at Village and National Levels in Lao PDR. Transbound Emerg Dis.

Perry, B.D., Gleeson, L.J., Khounsey, S., Bounma, P., 2002. The dynamics and impact of foot and mouth disease in smallholder farming systems in South-East Asia: a case study in Laos Background to the Laos case study. 21, 663-673.

Perry, B.D., Kalpravidh, W., Coleman, P.G., Horst, H.S., McDermott, J.J., Randolph, T.F., Gleeson, L.J., 1999. The economic impact of foot and mouth disease and its control in South-East Asia: a preliminary assessment with special reference to Thailand. Revue scientifique et technique (International Office of Epizootics) 18, 478-497.

Randolph, T.F., Perry, B.D., Benigno, C.C., Santos, I.J., 2002. The economic impact of foot and mouth disease control and eradication in the Philippines and control of the disease in the Philippines. Industrial Research 21, 645-661.

Rast, L., Windsor, P.a., Khounsy, S., 2010. Limiting the Impacts of foot and mouth disease in large ruminants in northern Lao People's Democratic Republic by vaccination: a case study. Transboundary and emerging diseases 57, 147-153.

Şentürk, B., Yalcin, C., 2005. Financial impact of foot-and-mouth disease in Turkey: acquisition of required data via Delphi expert opinion survey. Veterinarni Medicina 50, 451-460.

Şentürk, B., Yalçin, C., Senturk, B., Yalcin, C., 2008. Production Losses Due to Endemic Foot-and-Mouth Disease in Cattle in Turkey. Turk. J. Vet. Anim. Sci. 32, 433-440.

Tshering, P., 1995. An economic evaluation of the impact of foot and mouth disease and its control in Bhutan - VEERU-University of Reading.

Windsor, P.a., Freeman, P.G., Abila, R., Benigno, C., Verin, B., Nim, V., Cameron, a., 2011. Foot-and-Mouth Disease Control and Eradication in the Bicol Surveillance Buffer Zone of the Philippines. Transboundary and emerging diseases.

Young, J.R., O'Reilly, R.A., Ashley, K., Suon, S., Leoung, I.V., Windsor, P.A., Bush, R.D., 2014. Impacts on Rural Livelihoods in Cambodia Following Adoption of Best Practice Health and Husbandry Interventions by Smallholder Cattle Farmers. Transbound Emerg Dis.

Young, J.R., Suon, S., Andrews, C.J., Henry, L.A., Windsor, P.A., 2012. Assessment of Financial Impact of Foot and Mouth Disease on Smallholder Cattle Farmers in Southern Cambodia. Transboundary and emerging diseases.

Supplementary Tables

Table S1: Experts contacted to obtain published and unpublished work on the impact of FMD on smallholder farmers.

Expert	Institute	Area of work
James Young, Peter Windsor, S. Nampanya	University of Sydney	South-East Asia
Sarah Cleaveland, Tom Marsh	Glasgow University, Washington State University	Tanzania
Georgina Limon, Javier Guitan	The Royal Veterinary College, UK	South America
Andy Catley	Tufts, Feinstein International Centre	East Africa
Melissa McLaws	EuFMD-FAO	Central Asia
Henk Hogeveen	Wageningen University	Ethiopia
Rebecca Garabed	Ohio State University	Cameroon
Mark Bronsvoort	Roslin Institute	Cameroon
Steve Osofsky	Wildlife Conservation Society	AHEAD project, Southern Africa
Gavin Thomson	TADScientific	Southern Africa
Ferran Jori	CIRAD	Southern & Western Africa
Francois Maree	ARC-OVI	Southern Africa
Nick Lyons	The Pirbright Institute, EuFMD-FAO	Kenya
